# Morphometric analysis of the size-adjusted linear dimensions of the skull landmarks revealed craniofacial dysmorphology in *Mid1*-cKO mice

**DOI:** 10.1186/s12864-023-09162-2

**Published:** 2023-02-09

**Authors:** Yaohui Liang, Chao Song, Jieli Li, Ting Li, Chunlei Zhang, Yi Zou

**Affiliations:** 1 The Key Laboratory of Virology of Guangzhou, Jinan University, Guangzhou, China; 2 First Affiliated Hospital, Jinan University, Guangzhou, 510632 China; 3 Department of Biology, School of Life Science and Technology, Jinan University, Guangzhou, China

**Keywords:** *MID1*, Opitz syndrome, Craniofacial anomalies, Morphometrics, Procrustes superimposition, EMDA

## Abstract

**Background:**

The early craniofacial development is a highly coordinated process involving neural crest cell migration, proliferation, epithelial apoptosis, and epithelial-mesenchymal transition (EMT). Both genetic defects and environmental factors can affect these processes and result in orofacial clefts. Mutations in *MID1* gene cause X-linked Opitz Syndrome (OS), which is a congenital malformation characterized by craniofacial defects including cleft lip/palate (CLP). Previous studies demonstrated impaired neurological structure and function in *Mid1* knockout mice, while no CLP was observed. However, given the highly variable severities of the facial manifestations observed in OS patients within the same family carrying identical genetic defects, subtle craniofacial malformations in *Mid1* knockout mice could be overlooked in these studies. Therefore, we propose that a detailed morphometric analysis should be necessary to reveal mild craniofacial dysmorphologies that reflect the similar developmental defects seen in OS patients.

**Results:**

In this research, morphometric study of the P0 male *Mid1*-cKO mice were performed using Procrustes superimposition as well as EMDA analysis of the size-adjusted three-dimensional coordinates of 105 skull landmarks, which were collected on the bone surface reconstructed using microcomputed tomographic images. Our results revealed the craniofacial deformation such as the increased dimension of the frontal and nasal bone in *Mid1-*cKO mice, in line with the most prominent facial features such as hypertelorism, prominent forehead, broad and/or high nasal bridge seen in OS patients.

**Conclusion:**

While been extensively used in evolutionary biology and anthropology in the last decades, geometric morphometric analysis was much less used in developmental biology. Given the high interspecies variances in facial anatomy, the work presented in this research suggested the advantages of morphometric analysis in characterizing animal models of craniofacial developmental defects to reveal phenotypic variations and the underlining pathogenesis.

**Supplementary Information:**

The online version contains supplementary material available at 10.1186/s12864-023-09162-2.

## Background

The craniofacial development is one of the most complicate processes, which involve contributions from endoderm, mesoderm, ectoderm, as well as cranial neural crest cells (CNCCs) that are referred to as the ‘fourth germ layer’ [1]. The craniofacial structures are formed from the convergence of five embryonic tissue protrusions: the frontonasal processes (FNP), which developed into the lateral and medial nasal processes, a pair of the maxillary processes (MXP) and a pair of mandibular processes (MDP). Outgrowth of these processes is tightly orchestrated and largely occurs because of the emigration of highly proliferative neural crest cells which originate from the neural folds of the developing forebrain and midbrain [2]. Both mesoderm and the neural crest cells contribute to facial mesenchyme and eventually form the bone, cartilage, muscles, and connective tissues of the face [3].

Intrinsic cell defects and extrinsic environmental factors that dysregulate the CNCCs development can cause craniofacial malformations, which display as a range of characterized facial manifestations with orofacial clefts in severe situations [4]. Orofacial clefts averagely affect 1 in 700 live births and occur as an isolated malformation or as a part of syndromic anomalies [5]. Syndromic cleft lip and palate (CLP) are usually the common manifestations of congenital syndromes with various genetic defects and therefore, may have distinctive etiologies. Up to now, more than 350 potential candidate genes have been implicated to be associated with human orofacial clefts (OFCs) [6]. Rather than a single gene mutation, sporadic OFCs are often attributed to accumulated genetic variants that result in increased risks factors [7]. Although syndromic OFCs are more likely the syndromic consequences of defined genetic defects that usually involve loss-of-function mutations of precise genes, the genetic background and the existence of other modifying genes often have significant influence on the expression and penetrance of OFCs phenotype [8]. And therefore, failure in recapitulating the OFCs phenotype in transgenic mouse models with the genetic defects that have been proven in human OFC patients is not a rare situation. For instances, homozygote mutation in *PVRL1* causes an autosomal recessive CLP with ectodermal dysplasia and heterozygosity of the gene mutation is also a significant risk factor for non-syndromic CLP, while no cleft phenotypes have been observed in *PVRL1* null mutation mouse models [9, 10]. Mutations in the transcription factor *IRF6* are linked to isolated CLP, as well as to Van der Wounde syndrome (VWS), which is an autosomal dominant disorder associated with CLP [11]. However, *IRF6* knockout mice failed to display the full spectrum of the craniofacial manifestations seen in human cases. Instead, only cleft secondary palate along with defective tooth development and other minor craniofacial anomalies were displayed in *IRF6* null mutants [10]. On the other hand, some genes, e.g. *Bmpr1a*, encodes a receptor for Bmp4 and Bmp2, conditional knockout of which develop bilateral CLP in mice, was not implicated in human CLP linkage studies [12]. A detailed morphometric analysis of the phenotypic variability between primates and laboratory mice also revealed the evolutionary changes and differences in the pattern of facial modularity, based on the comparison of among-individual and interspecies correlations analysis of phenotypic-genetic covariation [13]. Despite of the highly conserved early craniofacial development observed in vertebrate, the variations of the phenotype between human and mouse reflect differences in the signaling mechanisms, genetic background, as well as in the cellular and developmental basis of craniofacial morphogenesis.

Opitz syndrome (OS) is a genetic heterogenous disorder characterized by developmental defects in midline structures, with *MID1* as the causative gene of X-linked OS [14]. Craniofacial anomalies were the most prominent feature of OS, with hypertelorism identified in almost every affected male patient, followed by anteverted nares and CLP, which were presented in about half of OS patients [14]. *MID1* gene encoded a microtubule-associated ubiquitin-E3 ligase and was believed to regulate early cellular events via mediating downstream target proteins turnover [15]. Recent studies suggested that MID1 was also presented as part of ribonucleoprotein complexes and promoted protein translation such as PDPK-1, BACE1, APP, and HTT [15–17]. Strong expressions of *MID1* in early developing facial prominence have been evidenced in both human and mouse [14, 18]. In chick, expression of *cMid1* was also observed in the cranial neural crest cells originated from rhombomeres 2 and regulated the neural crest cell migration, which made significant contribution to the cranial mesenchyme in BA1 and developed into maxillary and mandibular processes [19]. Previous studies demonstrated impaired neurological structure and function in *Mid1* knockout mice, while no CLP was observed [20]. However, given the highly variable severities of the facial manifestations observed in OS patients within the same family carrying identical genetic defects, subtle craniofacial malformations in *Mid1* knockout mice could be overlooked in these studies. Therefore, we propose that a detailed morphometric analysis should be necessary to reveal mild craniofacial dysmorphologies that reflect the similar developmental defects seen in OS patients. Furthermore, this approach may apply to the characterization of other craniofacial animal models that failed in displaying typical human phenotypes. Here, we generated a *Mid1* conditional knockout mouse line (Tg: *Mid1*^*flox/y*^; *Wnt1-Cre*), in which lose-of-function of *Mid1* was expected in neural crest derived tissues to avoid the convergence of morphological variation of cranial vault of *Mid1* null mice due to the potential change of neural development. A detailed morphological analysis of male P0 *Mid1*-cKO mice was performed using Generalized Procrustes analysis (GPA) of the size-adjusted three-dimensional coordinates of 105 skull landmarks that were identified on the 3D skulls reconstructed from microCT scan. Our results demonstrated the dysmorphology in the craniofacial regions derived from the FNP and MXP in the *Mid1-*cKO mutants, comparing with the control mice including their cre recombinase free counterparts, *Wnt1-Cre* transgenic mice and the ‘wild-type’ mouse of the same breed. The increased distance between the most posterior-medial point of the nasal bone and the anterior most point on the body of vomer, and the increased distance between the intersection of frontal process of maxilla with frontal and lacrimal bone and the most posterior-lateral point of the nasal bone in the *Mid1-*cKO mice were consistent with the most prominent facial anomalies of OS, including hypertelorism, prominent forehead, broad and/or high nasal bridge. Moreover, slightly increased head widths across the parietal bone were noticed in the *Wnt1-Cre* transgenic mice, which were reported with expansion of midbrain, along with other minor defects such as the increased proliferation in the developing inferior colliculus [21]. Given the difficulties in reproducing the human craniofacial anomalies in animal models due to the morphological differences, the work we presented here suggested the effectiveness of geometric morphometric analysis in phenotypic characterization of craniofacial animal models to uncover the potential genetic/cellular basis of pathogenesis.

## Results

### Validation of the ablated *Mid1* expression in *Mid1*-cKO male mice (Tg: *Mid1*^*flox/y*^; *Wnt1-Cre*)

**Fig. 1:**
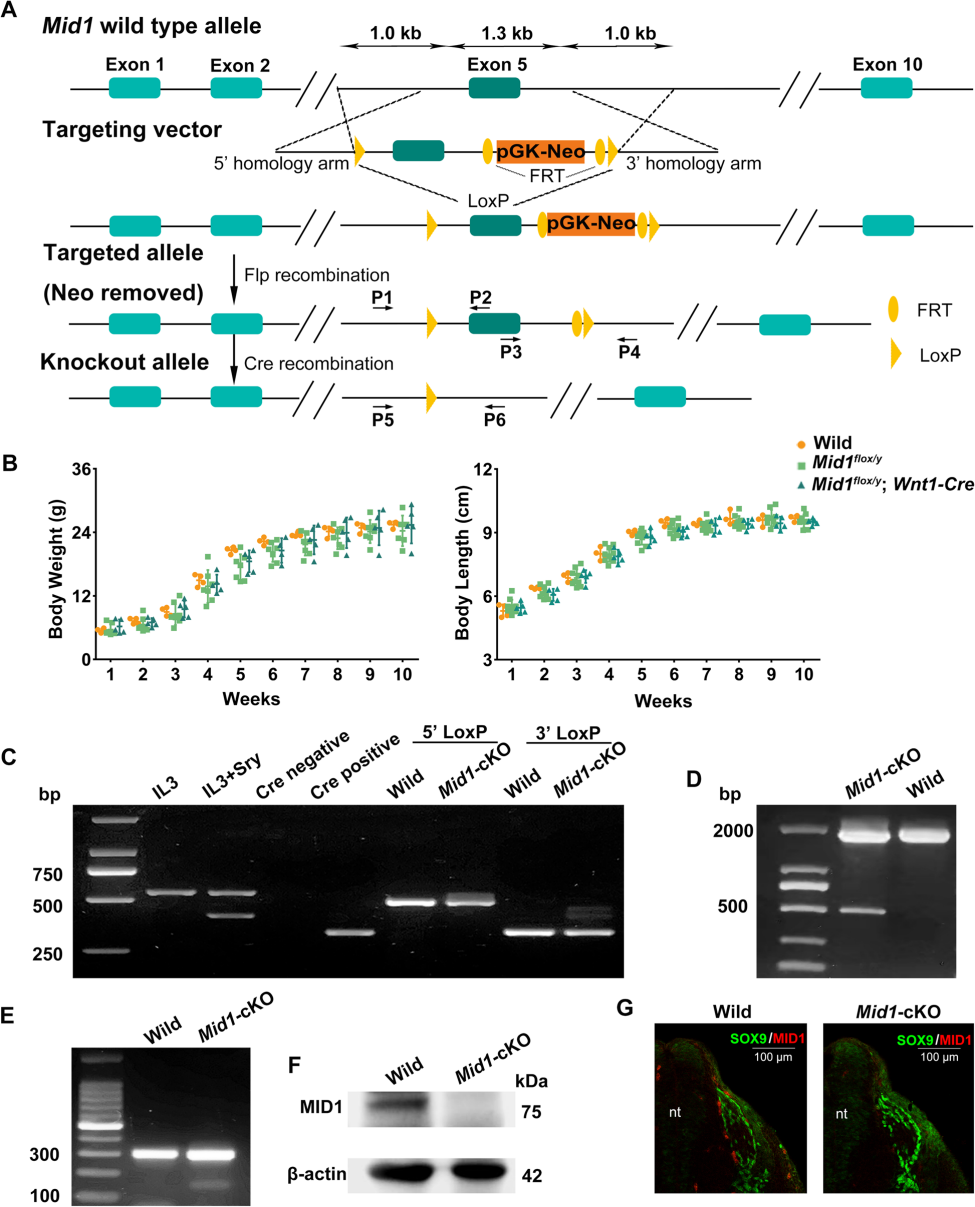
Generation of *Mid1* conditional knockout in NCCs. **A** Schematic diagram of the construction of the *Mid1*-floxed allele for generating the conditional knockout of *Mid1* in the NCCs. Exon 5 of *Mid1* was flanked by loxP sites. The FRT flanked Neo cassette was deleted by crossing chimeras with FLP females generated the *Mid1*-floxed F1 heterozygotes (*Mid1*^*flox/*+^), which were subsequently crossed with *Wnt1-Cre* transgenic mice to generate *Mid1*-cKO male mice (*Mid1*^*flox/y*^; *Wnt1-Cre*). The position and direction of primers used for genotyping were indicated with black arrows. **B** The body weight (left) and body length (right) have been monitored weekly in *Mid1*^+*/y*^, *Mid1*^*flox/y*^, and *Mid1*^*flox/y*^*; Wnt1-Cre* mice. **C** PCR genotyping. Fragments of *IL3* (544 bp) and *Sry* (402 bp, male-specific) were amplified for determination the gender of the mice. A 350 bp Cre replicon was amplified from the expression cassette of the Cre recombinase. Allele-specific PCR amplified 499 bp wild-type and 557 bp loxP fragments at the 5’ end loxP site to identify the F1 heterozygous. Allele-specific PCR amplified 344 bp wide-type and 466 bp loxP amplicons at the 3’ end loxP site to determine the heterozygosity at this locus. **D** PCR testing for identifying the deletion of *Mid1* exon 5. A 1721 bp wild-type and a 511 bp knockout fragments were amplified using genomic DNA of *Mid1*-cKO mice. **E** RT-PCR using the cDNA of a *Mid1*-cKO male mice, showing a 173 bp amplicon of the transcript from *Mid1* mutant allele in target tissue and a 310 bp amplicon of the transcript from intact *Mid1* allele. **F** Western blot detected a 75-kDa MID1 protein in wild-type male mice and a reduced expression in *Mid1*-cKO male mice. Protein levels were normalized with respect to the level of β-actin expression. **G** Immunofluorescence of transverse sections of neural tubes (nb) of E10.5 embryo showed the ablation of *Mid1* expression in the neural crest cells of *Mid1*-cKO mice. The migrating NCCs were identified with anti-Sox9 (green) and the expression of *Mid1* was identified with anti-MID1 (red)

The targeting vectors containing floxed *Mid1* exon 5 and the neomycin expression cassette were used for homologous recombination in mouse ES cells to generate the heterozygous *Mid1*^*flox/*+^ F1 mice (Fig. 1A). The Cre recombinase can catalyze intramolecular recombination between target recognition sequences (loxP sites) and resulted in an aberrant transcript of *Mid1*, which encodes a 306aa truncated *Mid1* with a frameshift mutation starting at 289aa. The *Mid1*-cKO mice are ostensibly healthy, grow to normal weight and body size, reproduce with typical 5 to 8 pup litters (Fig. 1B). *Wnt1-Cre* mediated KO of *Mid1* was obtained by breeding female *Mid1*-floxed mice with *Wnt1-Cre* transgenic mice and *Mid1*^*flox/y*^ Cre-positive mice were subjected to the following morphological analysis.

The sex, the presence of the expression cassette of the Cre recombinase and the loxP sites were verified by PCR genotyping (Fig. 1C). The *Wnt1-Cre* mediated knockout of *Mid1* exon5 in target tissue was confirmed by PCR of genomic DNA (Fig. 1D). Ablation of *Mid1* expression in NCC derivatives in the craniofacial mesenchyme of E10.5 *Mid1*^*flox/y*^ Cre-positive embryos was confirmed using RT-PCR, western blot, and immunofluorescence (Fig. 1E-G).

### Gross morphological analysis of bone and cartilage formation

**Fig. 2:**
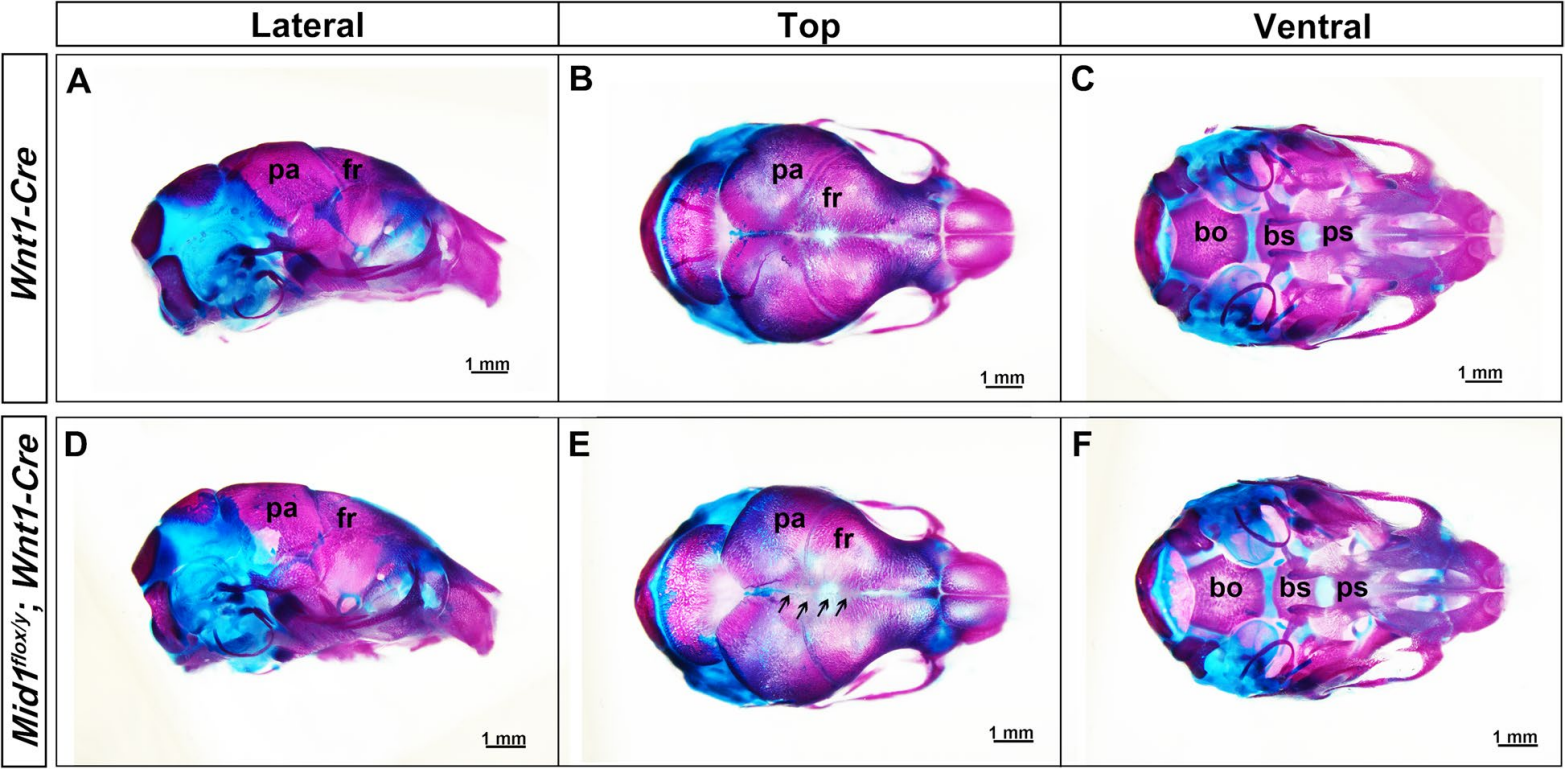
Wild-type and *Mid1*-cKO mouse skulls stained for cartilage and bone. The skulls of the P0 mice were stained with Alcian blue (cartilage) and Alizarin red (mineralization) after microCT scan, photographed from lateral (**A** and **D**), top (**B** and **E**), and ventral view (**C** and **F**). Hypo-ossification was noticed in the frontal bone close to the sagittal suture, indicated with black arrows (**E**)

We assessed the bone and cartilage formation in the skulls of P0 *Mid1*-cKO male mice on the completion of micro-CT. As shown by Alcian Blue and Alzarin red S staining, the skulls of *Mid1*-cKO mice displayed signs of hypo-ossification with severely decreased ossification identified in the frontal bone close to the intersection of sagittal suture and coronal suture (Fig. 2). Ectopic bone or cartilage were not observed.

### Overall morphometrics of the skulls

**Fig. 3:**
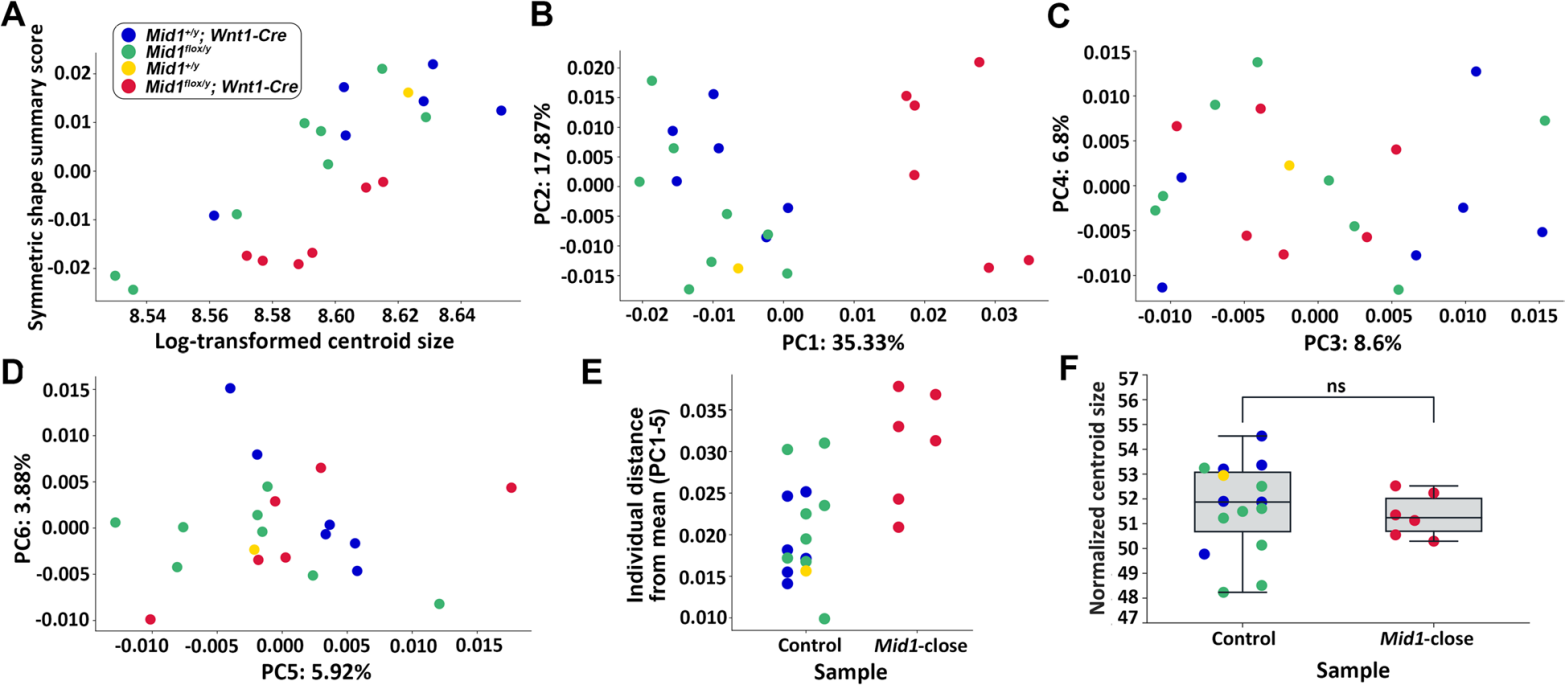
Basic morphometric analysis revealed the morphological variance of *Mid1*-cKO mice. **A** Linear association between the log-transformed centroid size of skull landmarks and the shape summary score achieved from the Procrustes coordinates in MorphoJ for each mouse. **B**-**D** Scatter plots of individual scores of PCA, displaying the degree of morphological variance, explained by percentages in the parentheses. Distribution of *Mid1*-cKO mutant mice (*Mid1*^*flox/y*^; *Wnt1-Cre*) and control littermates (*Mid1*^*flox/y*^, *Mid1*^+*/y*^*; Wnt1-Cre*, *Mid1*^+*/y*^) along the first six principal component axes, estimated using size-adjusted coordinates of 105 skull landmarks of each specimen. **E** The Euclidean distance between individuals and the grand mean shape of all specimens across PCs 1–5. **F** The normalized centroid sizes between the groups of control and *Mid1*-cKO mice showed no differences with statistical significance using unpaired two-tailed *t*-test (*P* > 0.05)

Testing of the consistency of the variation/covariation patterns before Procrustes fitting showed high correlation of shape matrices between *Mid1-*cKO and control mice (*r* = 0.9208, *p*-value < 0.001). The linear regression analysis was performed for both the symmetric component and asymmetric component of Procrustes coordinates on centroid size, respectively (Table S1 and S2). Our results displayed the strong linear association between the shape summary score and the log-transformed centroid size, indicating the significant attribution of size-associated shape variation caused by allometry (Fig. 3A). For symmetric components, 15.16% of shape summary score can be explained by log-transformed centroid size with statistical significance (*p*-value = 0.0036) using a permutation test for linear regression model with 10,000 randomization rounds (Table 1). Similarly, for asymmetric components, 7.12% of skull variation is size-associated (*P*-value = 0.0157). Although *Wnt1-Cre* mice displayed larger centroid size, no significant intergenic differences in skull size have been observed (Fig. 3A and F). PC analysis was conducted using size-adjusted Procrustes coordinate of symmetric component subsequently to evaluate the association between the craniofacial phenotypes and the genotypes. The first six PCs explained a cumulative 78.4% of craniofacial shape variance and the *Mid1*-cKO mice were well separated on the first axis that represented over a third of all variation, implicating distinctive facial features associated with the genetic defect (Fig. 3B-D, Table 2). This finding was further supported by the evidence of the morphological variations between each *Mid1*-cKO mouse and the grand mean shape of all samples, which was calculated as the average of the sum of scores across PCs 1–5 (PCs contributed > 5% of overall shape variances were counted) [22]. The differences between the sum of PC score of each *Mid1*-cKO mouse and the grand mean shape were greater than that between each of the control mice and the grand mean shape (Fig. 3E). It suggested that the craniofacial dysmorphology of *Mid1*-cKO mice was genotype specific and could not be completely explained by polymorphic variation between individuals. Calculation of the centroid sizes showed no significant difference between *Mid1*-cKO mice and the control mice, indicating the dysmorphology was primarily the consequence of shape variances (Fig. 3F).

**Table 1:** Permutation test for linear regression model

Component	Sum of square (SS)			% Predicted	*P*-value
	Total SS	Predicted SS	Residual SS		
Symmetric	0.01963642	0.00297624	0.01666018	15.16%	0.0036*
Asymmetric	0.00261780	0.00018637	0.00243143	7.12%	0.0592

*p < 0.005

**Table 2:** Variance of principle components for symmetric component

	Eigenvalue	% Var	% Cum	Control mean	*Mid1*-close mean	*P*-value
PC1	0.00029298	35.31	35.31	-0.008	0.024	< 0.001*
PC2	0.00014826	17.87	53.18	-0.002	0.004	0.319
PC3	0.00007145	8.61	61.79	0.001	-0.002	0.506
PC4	0.00005648	6.81	68.59	-0.001	0.001	0.987
PC5	0.00004914	5.92	74.52	-0.001	0.001	0.579
PC6	0.00003220	3.88	78.40	0.001	-0.001	0.822

*p < 0.001

The size-adjusted Procrustes coordination of the 105 skull landmarks of all samples were further integrated, considering both symmetric and asymmetric component, and were subjected to two-factor ANOVA using MorphoJ (Table 3). Significant differences in skull configurations were revealed on the symmetric component as well as on the DA of the asymmetric component (*p*-value < 0.0001) among samples. No significant asymmetric effect was found for FA, suggesting that the structural bias of was unlikely a result of fluctuation caused by small environmental perturbations.

**Table 3:** Procrustes ANOVA for symmetric and asymmetric component

Effect	SS	MS	df	*F*	*P*-value
Individual	0.00518780	0.0000330433	157	83.34	< 0.0001*
Side	0.00012615	0.0000008354	151	2.11	< 0.0001*
Ind * Side	0.00005987	0.0000003965	151	0.19	1.0000

*p < 0.0001

### Craniofacial dysmorphology of *Mid1*-cKO mice

**Fig. 4:**
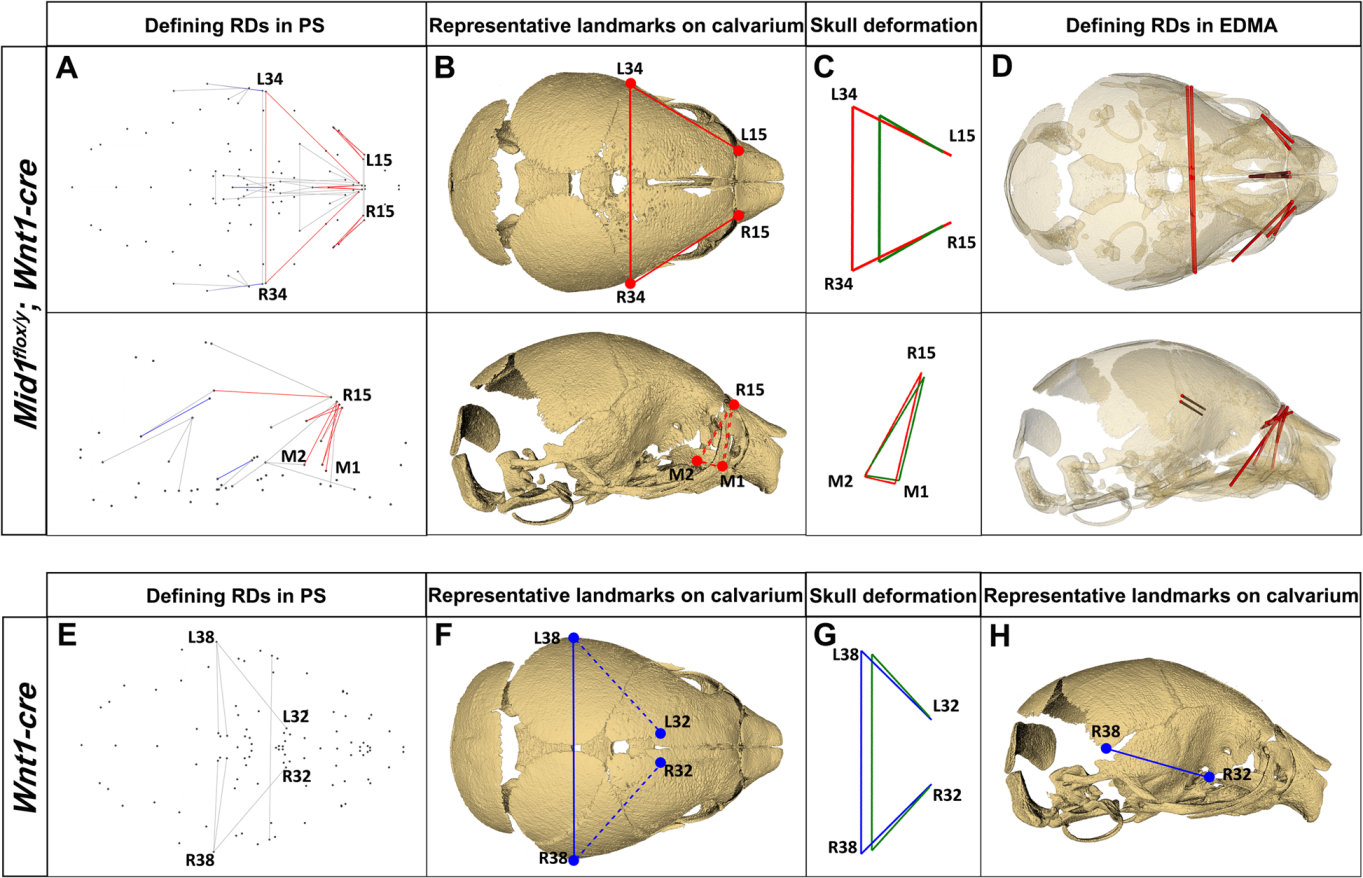
Morphological features of the skulls of *Mid1*-cKO mice and *Wnt1-Cre* transgenic mice displayed in 3D wireframes. **A** Defining relative linear dimensions (RDs) of *Mid1*-cKO mice that are always 5% larger (red) or smaller (blue) than the mean distances of the control mice (*Mid1*^*flox/y*^, *Mid1*^+*/y*^*; Wnt1-Cre*, *Mid1*^+*/y*^) are plotted from the superior (upper panel) and lateral view (lower panel), with the strongly differing RDs displayed in grey. **B** The microCT surface reconstructions of the correspondent *Mid1*-cKO mouse with the representative defining RDs elaborated on the top and lateral view, non-surface landmarks were illustrated in dotted lines. **C** Wireframe representing the mean shape differences between *Mid1*-cKO mice (red) and control mice (green) on selected landmarks on calvarium on the top and lateral view. **D** Defining RDs in EDMA analysis of *Mid1*-cKO mice that are always over 5% larger (red) than the mean distances of control mice were displayed on a representative transparent microCT skull on top (upper panel) and lateral view (lower panel). **E** The strongly different (> 2% larger than the means of control mice including *Mid1*^*flox/y*^ and *Mid1*^+*/y*^, displayed in grey lines) RDs of *Wnt1-Cre* mice plotted from the top view. **F** and **H** The microCT surface reconstructions of the correspondent *Wnt1-Cre* mouse with the representative strongly different RDs elaborated in blue lines on the top and lateral view, non-surface landmarks were illustrated in dotted lines. **G** Wireframe deformations represented the mean shape differences between *Wnt1-Cre* mice (blue) and control mice (green) on selected landmarks

To identify the characteristic differences of *Mid1*-cKO mice, the relative linear Euclidean distances (RDs) were derived from the symmetrical component of Procrustes superimposed size-adjusted landmark coordinates and in parallel, calculated using EMDA FORM analysis (Tables S3 and S5 and Figs. S1 and S3). The strongly differing relative linear dimensions (always identified larger or smaller in every individual *Mid1-*cKO mice, with the mean differences over 5% and *P* < 0.001) were annotated as the aberrant defining features of *Mid1* null mutants. Our results showed that among 1278 unique relative linear dimensions estimated from 105 skull landmarks and from 105 Procrustes superimposed coordinates, 20 RDs derived from PS landmark coordinates and 11 RDs derived from EMDA analysis reflecting the craniofacial configuration (e.g. nasal bone and pre-maxilla) were revealed to be the aberrant defining features of *Mid1*-cKO mice, elaborated with red (larger) and blue (smaller) lines respectively in the wireframe visualization (PS) and transparent 3D skull models (EMDA) from both inferior and lateral view (Fig. 4A-D, upper and lower panel). Except of the reduced length of the sphenoid and parietal bone, an overall elongated (anterior to posterior) craniofacial structures derived from embryonic FNP and MXP were noticed with *Mid1-*cKO mice in both PS and EMDA analysis, including the length of the frontal bone (R/L30-R/L34) and the height of the nasal bone (R/L2-M1). The RDs of the *Mid1*-cKO mice that strongly differed (over 5% larger or smaller, *p* < 0.001) from the mean of the control mice, but not consistently changed in every *Mid1*-cKO mouse, were recognized as the strongly differing RDs instead of defining features and illustrated in grey lines (Fig. 4A). The geometric morphometrics of cranial base were ostensibly normal for the *Mid1*-cKO mice. A remarkable consistency between the results of PS and EMDA analysis was displayed, with nine out of eleven defining features identified using EMDA were also recognized using PS landmark coordinates.

Interestingly, our results also demonstrated the slightly increased widths across the parietal for the *Wnt1-Cre* mice (2% larger than the mean of *Wnt1-Cre* negative mice, *P* < 0.01) (Table S4 and Fig. S2), in line with the expansion of the midbrain development shown in previous research [21]. The differing RDs of the *Wnt1-Cre* mice were illustrated in grey line in the 3D wireframe image from inferior and lateral view (Fig. 4E-H). Therefore, the expanded skull widths observed in the defining RDs in *Mid1-*cKO mice implicated complicate cumulative effects and could at least partially explained by the mild facial variations in *Wnt1-Cre* transgenic mice. The microCT surface reconstructions of the correspondent *Mid1-*cKO mouse and the *Wnt1-Cre* mouse are displayed from the top and lateral view (Fig. 4B, F, G). Deformations represented the mean shape differences between *Mid1-*cKO mice (red), *Wnt1-Cre* transgenic mice (blue), and control mice (green) on selected landmarks on calvarium were illustrated with wireframe images (Fig. 4C, G).

## Discussion

The facial morphology is highly variant interspecies and among individuals within the same species. The variations in skull morphology come from the intrinsic complexity of the craniofacial embryonic development that is coordinated by both genetic and environmental factors, as well as life-long postnatal facial morphological change, which can be affected by a broad range of issues, including the general physical health, diet, emotional stress. The early development of vertebrate face and the underline genetics are highly conserved, while interspecific variations have been observed in regarding to the origination of the cell population of CNCCs, the timing of neural crest cell migration, as well as in the tissue morphogenesis and interactions, etc. [23–25]. Therefore, it is barely possible to recapitulate the full spectrum of the phenotypes of human craniofacial anomalies in mouse models and CLP is uncommon in mouse mutants carrying the same genetic defects that have been ascribed to human ORFs. For instance, the expression of BMP4 is restricted in the ectoderm of the MNP, LNP, and MXP at the site of contact and the BMP signaling is essential for the outgrowth and fusion of these facial prominences. The null mouse mutant with defect in *Bmpr1b*, which encoded the Bmp type I receptor and was found associated with human CLP, failed to display cleft phenotype [12]. However, the mouse model bearing mutation in *Bmpr1a*, encoding another related Bmp type I receptor, displayed CLP with full penetration, while variations in *Bmpr1a* were not implicated in human CLP linkage studies [12].

The disagreement of the phenotypes was also observed in *Mid1* null mutant mouse model. *MID1* gene was first cloned at the breakpoint at Xp22 in an Opitz syndrome patient carrying an X-chromosome inversion and was subsequently identified as the causative gene in patients with X-linked Opitz syndrome [26]. Its mouse orthologue, *Mid1*, was the first gene found spanning the psuedoautosomal boundary on mouse X chromosome while its human counterpart subjected to X chromosome inactivation [27]. Previous studies of *Mid1*-null mice revealed abnormal brain development and defects in motor coordination, while failed to phenocopy any of the facial manifestations seen in OS patients. We generated a *Mid1*-cKO mouse line that expressed a truncated form of MID1 of 306 amino acids with a frameshift mutation starting from aa289 in NCCs. It was a loss-of-function mutation since the variant lost the B-Box C-terminal domain, FN3 and SPRY, which were essential for its microtubule-association and protein interactions. Given the intact B-boxes domain in the truncated MID1, it might play a dominant-negative role by tethering Alpha4, the regulatory subunit of PP2Ac and was involved in the pathogenesis of OS [28]. A total number of six knockout male mice were obtained from mating of nine F1 *Mid1*^*flox/*+^ female and four *Wnt1-Cre* transgenic male. Their siblings of eight Cre-negative floxed male mouse litters, one *Mid1*^+*/y*^ male litter, and six *Mid1*^+*/y*^ Cre-positive mice were used as controls in morphometric analysis. The Principal Components analysis quantified the skull shape variations of all the mice along major axes and revealed the distinctive overall morphometrics of *Mid1-*cKO mice on the first major axes. The craniofacial regions that displayed dysmorphology associated with *Mid1* null mutation were identified using size-adjusted Procrustes superimposed landmarks, as well as determined with quantified CS-adjusted linear distances that contributed to shape differences using EDMA FORM analysis. Of particular interest, the results of GPA analysis revealed significant directional asymmetry in the skull configurations of our samples, although no features of left–right asymmetry had been reported in OS patients. Whether the directional asymmetry is genotype-specific and a result of defective *Mid1* needs to be clarified with further morphometric analysis on the asymmetric components as well as with functional studies. As asymmetric expression of *cMid1* on the right side of Henson’s node and a key role in establishing left–right axis have been demonstrated in chick embryos, the lack of laterality defects in human patients with *MID1* mutation implicated the existences of other modifying genes, e.g., *MID2*, that play compensatory roles [29, 30]. Although no cleft lip/palate was observed in our *Mid1-*cKO mice, the strongly differed craniofacial structures observed in *Mid1-*cKO mice such as expanded frontal and nasal bone were derived from embryonic FNP/MXP, and in line with the most prominent facial features including hypertelorism, prominent forehead, broad and/or high nasal bridge in OS patients. Reduced cranial vault parameters (~ 8–9% reduced length of the sphenoid and parietal bone) were also observed, possibly in compensation to the elongated craniofacial structures to maintain the overall skull shape or actually reflecting the underlining defects in the neural development that was also reported in *Mid1* mouse mutants as well as in OS patients [14, 20]. The two approaches displayed high consistency in characterizing the mutant phenotype, likely due to the well separated size and shape changes because of the high spatial resolution rendered with densified landmarks. Recent research suggested the landmark-free morphometrics for high-resolution phenotyping, which displayed high efficiency in characterizing craniofacial anomalies without intensive labor involved in landmark annotation [31]. Instead of struggling to phenocopying the human manifestations in animal disease models, the work presented in this study, along with those of the others, suggested that the morphometric analysis, which has been extensively used in evolutionary biology and anthropology, could be a valuable tool in characterizing developmental morphologies that display low intergenic consistency [32–37]. Our findings suggested that a detailed morphometric analysis could help to interpret the phenotypic variation and to highlight the pathogenesis when identical features seen in human defects were difficult to recapitulate in animal models.

## Conclusion

Given the high interspecies variances in craniofacial development, researchers often find difficulties in the attempts to recapitulate the identical human facial anatomy in animal models of craniofacial development. Our work presented in this study revealed the craniofacial deformation such as the increased dimension of the frontal and nasal bone in *Mid1*-cKO mice using both GPA and EMDA analysis, in line with the most prominent facial features such as hypertelorism, prominent forehead, broad and/or high nasal bridge seen in OS patients. While extensively used in evolution biology, our results suggested the great potential of morphometric studies in characterizing animal models of craniofacial developmental defects to reveal phenotypic variations and the underlining pathogenesis.

## Methods

### Reagents

Agarose, Tween-20, Triton X-100, Ammonium Persulfate (AP), Acrylamide/Bis-acrylamide, 30%solution, TEMED and 4% paraformaldehyde were from Sigma-Aldrich Co. (St. Louis, MO, USA). Skim Milk Powder was purchased from FUJIFILM Wako Pure Chemical Corporation (Chuo-Ku, Osaka, Japan, 190–12,865). Protein sample Loading Buffers and PageRuler™ Prestained Protein Ladder were from Thermo Fisher Scientific (Waltham, MA, USA). DNA molecular weight markers were from Takara Bio, Inc. (Kusatsu, Shiga, Japan). Bovine Serum Albumin (BSA) was from Genview (Calimesa, CA, USA).

### Generation of conditional *Mid1*-knochout mice

The targeting vector containing 1.0-kb-long homology arms at the 5′ and 3′ end of *Mid1* exon 5 was used to generate *Mid1*-floxed allele with neomycin selection. The loxP/FRT flanked Neo cassette (driven by the PGK promoter) was inserted on the 3′ end of exon 5 and a single loxP site was inserted at the 5′ end of exon 5 (Fig. 1A). The targeting vector was electroporated into JM8A3 ES cells, a C57BL/6 derived mouse embryonic stem cell line. The recombinant ES cell clones were identified by neomycin resistance and PCR screening, followed by microinjection into blastocysts of C57BL/6 and implantation in pseudo-pregnant females to generate chimeras. The males were crossed to FLP females (Jackson Laboratory) to delete the FRT flanked Neo cassette and generate the F1 heterozygotes. Female *Mid1-*floxed mice (*Mid1*^*flox/*+^) were then crossed with *Wnt1-Cre* transgenic mice (Jackson Laboratory) to generate the conditional *Mid1* knockout male mice (*Mid1*^*flox/y*^; *Wnt1-Cre*, hereafter *Mid1*-cKO). All mice were bred and housed in the Laboratory Animal Management Center of the Jinan University in a pathogen free facility. Embryos were considered 0.5 days old when a vaginal plug was observed in the morning after mating. Mouse body weight and body length were measured every week from 1 to 10 weeks of age for mouse strains. Mice were anesthetized using 2% isoflurane for photos being taken with a fixed Kodak DC290 digital camera (Eastman Kodak Co., Rochester, NY, USA). The images were used for measuring the distances from the tip of the nose to the end of the tail with ImageJ by the same observer. After the measurement, these anaesthetized mice were euthanized. The mice we used in our study were euthanized with CO_2_ gas prior according to the American Veterinary Medical Association (AVMA) Guidelines for Euthanasia of Animals (2020). The animals were decapitated at approximately 5 min after the induction of CO_2_ without anesthetics, CO_2_ was led into the cage by a tube at a displacement rate of 30% of the chamber volume with carbon dioxide per minute, CO_2_ flow was maintained for at least 1 min after respiratory arrest. *Mid1*^*flox/*+^ mice were constructed by Nanmo Biotechnology Co., Ltd. (Shanghai, China), Wnt1-Cre transgenic mice were purchased from Jackson Laboratory (Bar Harbor, ME, USA) and C57BL/6 J mice were ordered from The Experimental Animal Center of Guangdong (Guangzhou, China). 37 pup and 6 mouse embryos were used in this study, all of them are male. Specifically, 6 mouse embryos were used to confirmed ablation of *Mid1* expression by RT-PCR, western blot, and immunofluorescence, we chose a small sample size to confirm ablation of *Mid1* expression as it was enough to assess whether conditional *Mid1*-knockout mice were generated in the present study. Then, based on previous studies, measured the body weight and body size of 16 pup to identify the growth and the skulls of 21 P0 pup were subjected to micro-computed tomography scanning [38].

### PCR genotyping of heterozygous F1 and conditional *Mid1* knockout mice

Genomic DNA was extracted from the tail tip using the Tissue DNA Kit (Omega, Norcross, GA, USA, D3396-02), according to the manufacturer’s instructions. The primers for PCR genotyping are listed in Table 4. The position and direction of primers were illustrated on the diagram in Fig. 1A. The sexes of the cubs were determined by PCR amplification of *IL3* (544 bp) and *Sry* (402 bp). Primer pair P1 and P2 were screen primers used identify the insertion of 5’ end loxP in exon 5, 557 bp amplicon with loxP and 499 bp amplicon without loxP, respectively. Primer pair P3 and P4 were used to identify the correct insertion of the 3’ end loxP in exon 5, 466 bp amplicon with loxP and 344 bp amplicons without loxP. The presence of the expression cassette of the Cre recombinase was identified with the primer Wnt1-Cre-F and Wnt1-Cre-R, which amplified 350 bp fragments from Cre transcripts. PCR was performed in a total volume of 20 µl at 94 °C 2 min (94 °C 30 s, 57 °C 30 s, 72 °C 1 min) for 30 cycles, and ending at 72 °C for 2 min using Premix Taq™ DNA Polymerase (Takara, RR003A). Primer pair P5 and P6 were used to verify that the deletion of *Mid1* exon 5, 1721 bp amplicons for the wild-type allele and 511 bp amplicons for the knockout allele, respectively. PCR was performed in a total volume of 20 µl at 94 °C 2 min (94 °C 30 s, 60 °C 30 s, 72 °C 2 min) for 30 cycles, and ending at 72 °C for 2 min using Premix Taq™ DNA Polymerase (Takara, RR003A). 5 μl PCR products were resolved by DNA electrophoresis on 1–3% agarose gel and visualized under UV light using FluorChem imaging system (Alpha Innotech Inc., CA, USA).

**Table 4:** The primers for PCR genotyping

Primer name	Primer sequences (5'-3')	Size (bp)
IL3-F	GGGACTCCAAGCTTCAATCA	544
IL3-R	TGGAGGAGGAAGAAAAGCAA	
Sry-F	TGGGACTGGTGACAATTGTC	402
Sry-R	GAGTACAGGTGTGCAGCTCT	
Wnt1-Cre-F	CAGCGCCGCAACTATAAGAG	350
Wnt1-Cre-R	CATCGACCGGTAATGCAG	
P1	GGCACATTTCAGACACCAGAG	WT: 499
P2	CTGTAGGTGTGAATTCGAGGC	*Mid1*-cKO: 557
P3	CAGTTAGTCAAGGAAAAGCATCAC	WT: 344
P4	GAGCATTTAAATTGTATCCTGTCA	*Mid1*-cKO: 466
P5	GGCACATTTCAGACACCAGAG	WT: 1721
P6	TGCCAAAATACGGACGAACCAT	*Mid1*-cKO: 511

### RNA extraction and reverse transcription of validation of truncated *Mid1* expression

Total RNA was isolated from the E10.5 embryonic craniofacial prominences using Trizol reagent (Invitrogen, Carlsbad, CA, USA, 15,596,018). Reverse transcriptase-polymerase chain reactions (RT-PCR) were conducted with 1 μg of total RNA for each sample using the PrimeScript® RT reagent kit with gDNA Eraser (TaKaRa, RR047A) according to the manufacturer's instructions. The forward (5′- AATCATTCAGCAACGAAGACA -3′) and the reverse (5′- CGGGAGAAGAAACTGCTAGAA -3′) primers spanning *Mid1* exon 5 were used to amplify the cDNA of wild-type (310 bp) or truncated (173 bp) *Mid1* transcripts. PCR was performed in a total volume of 50 µl at 94 °C 2 min (94 °C 30 s, 55 °C 30 s, 72 °C 20 s) for 35 cycles, and ending at 72 °C for 2 min using Premix Taq™ DNA Polymerase (Takara, RR003A). 5 μl PCR reactions for each sample were resolved on 2% agarose gel and visualized under UV light using FluorChem imaging system (Alpha Innotech Inc., CA, USA).

### Protein extraction and western blot analysis

Tissue of E10.5 embryonic craniofacial prominences was homogenized in 30 μl RIPA lysis buffer (Sigma-Aldrich, R0278) plus protease inhibitor cocktail (Sigma-Aldrich, P8340). The protein concentration was quantified using the BCA kit (Thermo Scientific, 23,225). 30 μg of total proteins for each sample were electrophoresed on 10% SDS-PAGE, followed by electro-transferring to a polyvinylidene fluoride (PVDF) membrane, as described in our previous publication [39]. Membranes were blocked with 5% skimmed milk in TBST (0.05% Tween 20) for 2 h, followed by incubation overnight at 4 °C with a rabbit anti-MID1 polyclonal antibody (1:500, Abcam, Cambridge, MA, USA, ab70770) or a mouse anti-β-actin monoclonal antibody (1:10,000, ProteinTech, Rosemont, IL, USA, 66,009–1). After several washes, membranes were then incubated for two hours with an HRP-conjugated goat anti-rabbit or an HRP-conjugated goat anti-mouse IgG antibody, respectively (1:3000, ProteinTech, SA00001-1, SA00001-2) at 4 °C. Immunoreactive bands were visualized by chemiluminescence using the Immobilon Western Chemiluminescent HRP substrate kit (Millipore, Billerica, MA) and developed in an Amersham Imager 680 (GE Healthcare Bio-Sciences, Pittsburgh, PA, USA).

### Immunohistochemistry and immunofluorescent staining

E10.5 mouse embryos were collected in ice-cold PBS and fixed in 4% paraformaldehyde at 4◦C overnight. The embryos were transferred to 5% sucrose in PBS at 4◦C until embryos sink and then dehydrated in 30% sucrose in PBS at 4◦C overnight. The dehydrated embryos were embedded in 50:50 30% sucrose/PBS: OCT compound (Sakura, Torrance, CA, USA, 4583) and stored at –80 °C before use. 10 μm sections were sliced from cryoembedded blocks from the indicated orientation using a freezing microtome (Model CM3050; Leica Microsystems, Bannockburn, IL, USA) and mounted on glass slides. Sections were washed in PBT (0.1% Triton X-100), blocked for 1 h in 10% BSA/PBT at room temperature, then incubated with the indicated primary antibodies, rabbit anti-MID1 polyclonal antibody (1:500, Abcam, ab70770) and mouse anti-SOX9 (1:400, Abcam, ab76997), at 4 °C overnight, respectively. After three times washes, immunohistochemistry was conducted on sections using the Alexa Fluor 488-AffiniPure Goat Anti-Mouse IgG (H + L) and Alexa Fluor® 594 AffiniPure Goat Anti-Rabbit IgG (H + L) secondary antibodies (1:1000, Jacksons ImmunoResearch, West Grove, PA, USA, 115–545-003, 111–585-003). Finally, the slides were washed three times with PBT and were then incubated with Hoechst 33,342 (1 μg/mL, Sigma-Aldrich, B2261) for nuclei staining for 15 min and mounted in Fluoromount™ Aqueous Mounting Medium (Sigma Aldrich, F4680-25 mL) for 30 min. The images with 4.19 megapixels were captured on Olympus FV3000 confocal microscope (Olympus Corporation, Tokyo, Japan) using FV3000 Galvo Scan Unit at 20 × magnification and processed with FV31S-SW Viewer software (version 2.5).

### Micro-CT imaging and 3D reconstruction

The heads of P0 mice were dissected after sacrifice and were fixed in 4% paraformaldehyde for 1 to 3 days. The skulls of P0 mice were subjected to micro-computed tomography scanning using a SkySkan 1276 small animal micro-CT scanner (Burker, Kontich, Belgium) and the scanning was performed at each 0.2 degree for a full 360° rotation around the longest axis of the specimen. Micro-computed tomography images of all P0 skulls were obtained at 650 ms exposure at 55 kV 200 μA, and 6 μm resolution. Two-dimensional projection images were reconstructed from averaging two frames using the manufacturer’s standard postprocessing procedures. Three-dimensional voxel renderings of the cross-sectional image stacks were completed using the free online tool 3D Slicer (http://www.slicer.org). The segmentation threshold ranges from 80 ± 1 to 255 was set within 3D Slicer to define the bone surfaces from soft tissue. Median smooth method and standard deviation of 3.0 mm was applied to smooth the skull models using segment editor in 3D Slicer [40].

### Bone and cartilage staining

To remove the scalp, the mouse heads were scalded in hot water (70 °C) for 30 s on the completion of microCT scan. The skulls were then fixed in 95% ethanol overnight, followed by overnight treatment at room temperature in acetone to remove fat. The samples were briefly washed and stained for cartilage in alcian blue (Alcian Blue 8GX, Sigma-Aldrich, A5268) for 24 h. The samples were then transferred to 70% ethanol for 6–8 h, followed by incubation in 1% potassium hydroxide until the tissues became visibly clear. The bone was counterstained in alizarin red stain solution (Alizarin Red S, Sigma-Aldrich, A5533) overnight. The samples were again cleared in 1% potassium hydroxide/20% glycerol for 2 days and then stored in glycerol: ethanol (1:1) before imaging. High-definition 5.9-megapixel images were captured under a Nikon stereomicroscope (SMZ18, Chiyoda-ku, Tokyo, Japan) with SHR Plan Apo 1 × objective using a Nikon's DS-Fi3 microscope camera and processed with NIS-Elements software (version 5.10).

### Morphometric analysis

**Fig. 5:**
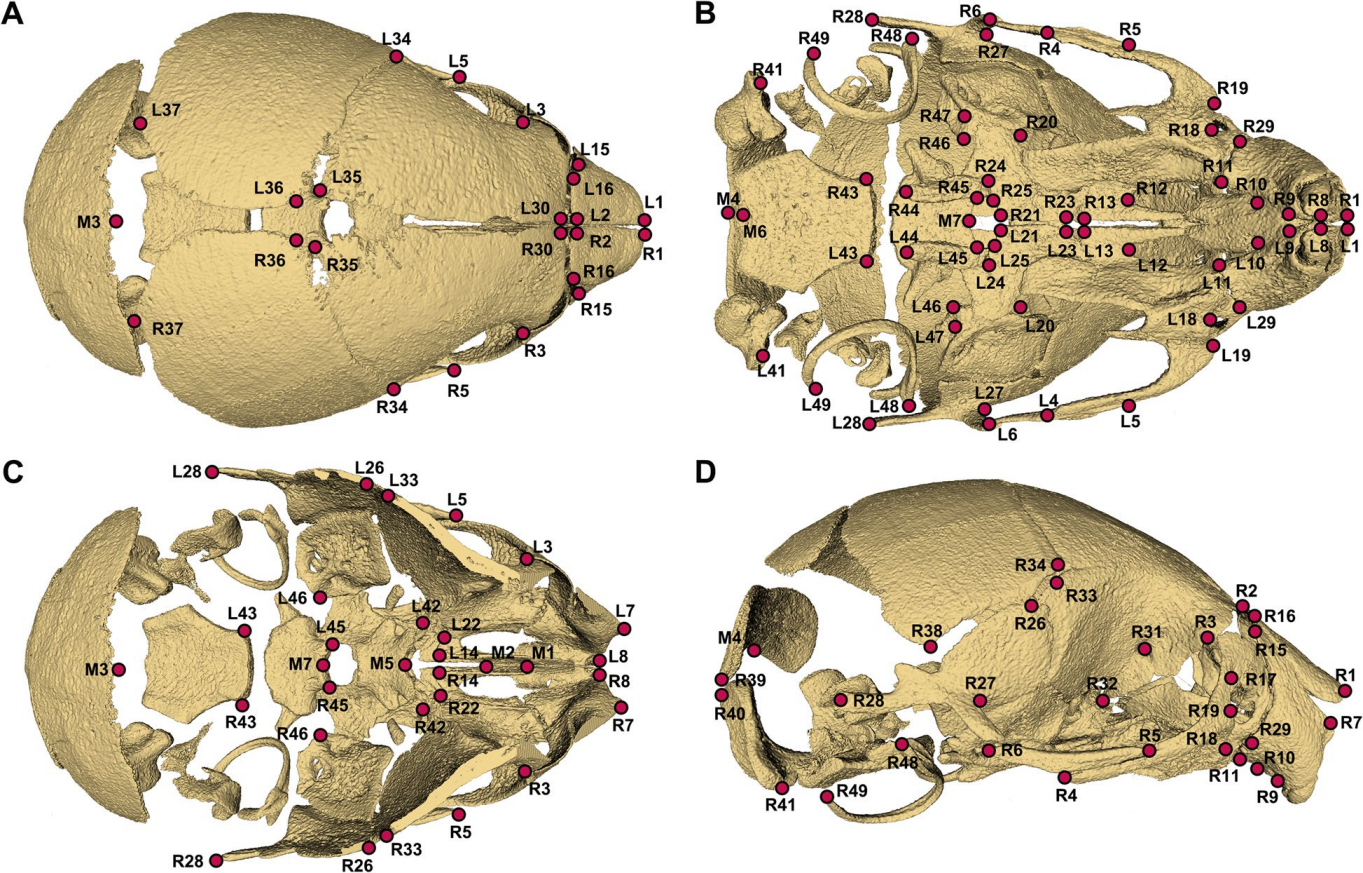
Anatomic locations of the 105 landmarks (red colored dots) on the 3D reconstruction of a microCT scan of a male P0 *Mid1*-cKO mouse, identified on the superior (**A**), inferior (**B**), superior with the calotte removed (**C**), and the lateral view. Midline landmarks start with M and bilateral landmarks start with L&R

The geometric dataset used in this study comprised the Cartesian coordinates of 105 landmarks representing the overall morphology of the skulls of 21 P0 male F2, which were the offspring from mating 9 F1 heterozygous females (*Mid1*^*flox/*+^) and 4 *Wnt1-Cre* males (Table S6). These 21 P0 male F2 consist of 6 *Mid1*-cKO mice (*Mid1*^*flox/y*^*; Wnt1-Cre*), 6 *Wnt1-Cre* mice (*Mid1*^+*/y*^*; Wnt1-Cre*), 8 floxed mice (*Mid1*^*flox/y*^) and 1 wild-type mice (*Mid1*^+*/y*^). These landmarks were chosen both to represent the overall morphology of the skull with the enhancement on the facial region and so that they could be precisely digitized due to their repeatability (Fig. 5 and Table S7) (https://getahead.la.psu.edu/landmarks) [41, 42]. Three-dimensional coordinates of these 105 landmarks were annotated on the 3D skull twice by a single observer, who was blind from the sample genotypes (Table S8). The errors were unbiasedly distributed on all axes and the error averages were less than 0.02% of centroid sizes for all samples [43]. The Euclidean distance matrix was reconstructed with the inter-landmark distances adjusted by centroid size using “panda” and “numpy” in python [44–48]. The morphological variations were evaluated using Euclidian distance matrix analysis (EDMA) with the formular described by Lele and Richtsmeister [49], as well as using the linear distance calculated from the size-adjusted coordinates derived from Generalized Procrustes analysis (GPA) [50–52]. The morphological differences between mice indicated the relative changes in relation to the entire geometry of the skull, rather than the changes in absolute measurements. The cranial bones in this study were divided into three modules defined by distinctive functional and developmental organizations, the cranial base formed by endochondral ossification, the cranial vault formed by intramembranous ossification and predominantly affected by neural development, and craniofacial structures mainly affected by muscle development [13]. Given the likelihood of compensatory change across modules to maintain an overall cranial morphology, the lineal dimensions were recorded only between landmarks within the same module, as described by Percival et al. [22]. As the result, a set of 1278 elements consisted of the Euclidean distances between landmarks on the same side and within the same modules in the matrix of bilateral measures of 105 landmarks were withdrawn and summarized as a vector [52]. The form vectors were then size adjusted with scaling factors of centroid size before locating shape differences [44, 53].

Geometric morphometric (GM) analysis involving Generalized Procrustes analysis (GPA) to superimposed landmark configurations of all shapes were performed using MorphoJ (v1.0.7a) [54]. All landmark configurations were transformed to Procrustes coordinates with the process of Procrustes superimposition (PS) by “New Procrustes fit” in MorphoJ. The sum of the squared deviations (m^2^) was calculated followed by nonparametric permutation test using the vegan package of R to evaluate the variance contribution between any two mice within *Mid1-*cKO group, as well as between *Mid1-*cKO mice and the control mice. *P* < 0.05 indicated no overfitting and all landmarks were included in the following morphometric analysis [13, 55–57].

The 3D skeletal landmark configurations in this study were proposed to be object symmetry and therefore, the mirror configurations were created before Procrustes superimposition. Components of symmetric and asymmetric shape variation were exacted from MorphoJ. Allometric multivariate regression on logarithm of centroid size was carried out with “Regression” in MorphoJ on the symmetric and asymmetric component (Tables S1 and S2), respectively, to exclude the size-associated shape variation, which was not considered to be pivotal in the dysmorphology of *Mid1*-cKO mice since developmental delay was not reported in either OS patients or *Mid1* null mutant mice [14, 20, 58]

The significance of variations in landmark coordinates were evaluated for both symmetric and asymmetric components using Procrustes ANOVA. Symmetric components were computed as the means of mirror configurations and original configurations. The asymmetric components were quantified as the differences between the original and reflected mirror configurations within individuals [59]. Directional asymmetry (DA) and fluctuating asymmetry (FA) were revealed in measuring the level of asymmetry. The average differences between left and right were quantified, and DA was referred to the systematical changes on one side, while FA represented random developmental differences due to the developmental instability [59]. The asymmetric effect in the pooled samples was evaluated by two-factor, mixed-effect ANOVA on the size-adjusted asymmetric components [60].

Principal component analysis (PCA) was implemented to quantify and visualize the shape variation of each individual mouse along the major axes and the relative genetic contribution. The association between genetic composition and craniofacial morphology was evaluated with Principal Components Analysis (PCA) using the symmetric component of size-adjusted landmark configurations. The difference of the principal component (PC) scores on between *Mid1-*cKO mice and the control mice were tested for significance using unpaired two-tailed *t*-test [43]. The results of PCA were visualized with the ‘sklearn’ machine learning module in Python (v3.8.7).

### Relative linear dimensions (RDs)

The inter-landmark distances between symmetrical component of Procrustes superimposed size-adjusted landmark coordinates (regression residuals) as well as the size-adjusted Euclidean distances in EMDA were calculated using Python and were referred as the relative linear dimensions (RDs), which reflected the relative linear dimensions rather than the absolute distances of morphological forms [22]. Given the distinctive tissue origins at various developmental stages, the skulls were identified as three modules, including facial, cranial vault, and cranial base [1]. RDs were calculated within each module and within the same side. Cross-side measures of the Euclidean distances were only recorded between two mirror landmark coordinates (Tables S3, S4 and S5). Defining features of *Mid1*-cKO mice were recognized as the RDs of every individual knockout mouse that were always larger or smaller (5%) than those of the control individuals. Strongly differing RDs were identified as the significantly different mean measures of Euclidean distances (5% larger or smaller, *P* < 0.001) between the *Mid1*-cKO mice and the control mice using unpaired two-tailed student’s *t*-test (*p*-value < 0.05).

## Supplementary Information

**Additional file 1: Fig S1.** 3D wireframe plot of one *Mid1* nullmutant mouse, showing the aberrant defining features in red/blue lines(identified larger or smaller in every individual *Mid1*-cKO mouse, with >5%mean difference and *P*<0.001) and the strongly differing features in greylines (over 5% mean difference and *P*<0.001, but not always identified inevery individual *Mid1*-cKO mouse).

**Additional file 2: Fig S2.** 3D wireframe plot on one *Wnt1-Cre *transgenic mouse, showing the strongly differing features in grey lines (over2% mean difference and *P*<0.01), comparing with their Cre-negative littermates.

**Additional file 3: Fig S3.** 3D wireframe plot on one *Wnt1-Cre *transgenic mouse (EDMA).

**Additional file 4: Fig S4.** Original figures of gel and blot.

**Additional file 5: Table S1.** Symmetric Regression Residualsextracted from MorphoJ for allometric correction.

**Additional file 6: Table S2.** Asymmetric Regression Residualsextracted from MorphoJ for allometric correction.

**Additional file 7: Table S3.** RDs calculated using size-adjustedlandmark coordinates of symmetric Regression Residuals for *Mid1*-cKO mice.

**Additional file 8: Table S4.** RDs calculated using size-adjustedlandmark coordinates of symmetric Regression Residuals for *Wnt1-Cre* transgenicmice.

**Additional file 9: Table S5.** CS-normalized Inter-landmark distancesof *Mid1*-cKO mice used in EDMA analysis.

**Additional file 10: Table S6.** Breeding schemes.

**Additional file 11: Table S7.** Skull landmarks used in morphometricanalysis.

**Additional file 12: Table S8.** Raw data of landmark coordinatescollected on the skull surfaces reconstructed from microCT scan images using 3DSlicer.

## Data Availability

All data generated or analyzed during this study are included in this published article [and its supplementary information files].

## Abbreviations

EMT: Epithelial-mesenchymal transition
CNCCs: Cranial neural crest cells
FNP: Frontonasal processes
MXP: Maxillary processes
MDP: Mandibular processes
LNP: Lateral nasal processes
MNP: Medial nasal processes
MxP: Maxillary processes
CLP: Cleft lip/palate
OFCs: Orofacial clefts
VWS: Van der Wounde syndrome
OS: Opitz syndrome
AP: Ammonium persulfate
BSA: Bovine serum albumin
NIH: National Institutes of Health
PVDF: Polyvinylidene fluoride
EDMA: Euclidean distance matrix analysis
GPA: Generalized Procrustes analysis
GM: Geometric morphometric
PS: Procrustes superimposition
CS: Centroid size
DA: Directional asymmetry
FA: Fluctuating asymmetry
PCA: Principal component analysis
PC: Principal component
RDs: Relative linear dimensions
