# Supplementary material for: Morphometric analysis of the size-adjusted linear dimensions of the skull landmarks revealed craniofacial dysmorphology in Mid1-cKO mice
Source: BMC Genomics. 2023 Feb 9;24:68. doi: 10.1186/s12864-023-09162-2 (PMC9912615; doi:10.1186/s12864-023-09162-2)
Supplement: Supplementary file 4 — Additional file 4: Fig S4. Original figures of gel and blot. [file 12864_2023_9162_MOESM4_ESM.docx]

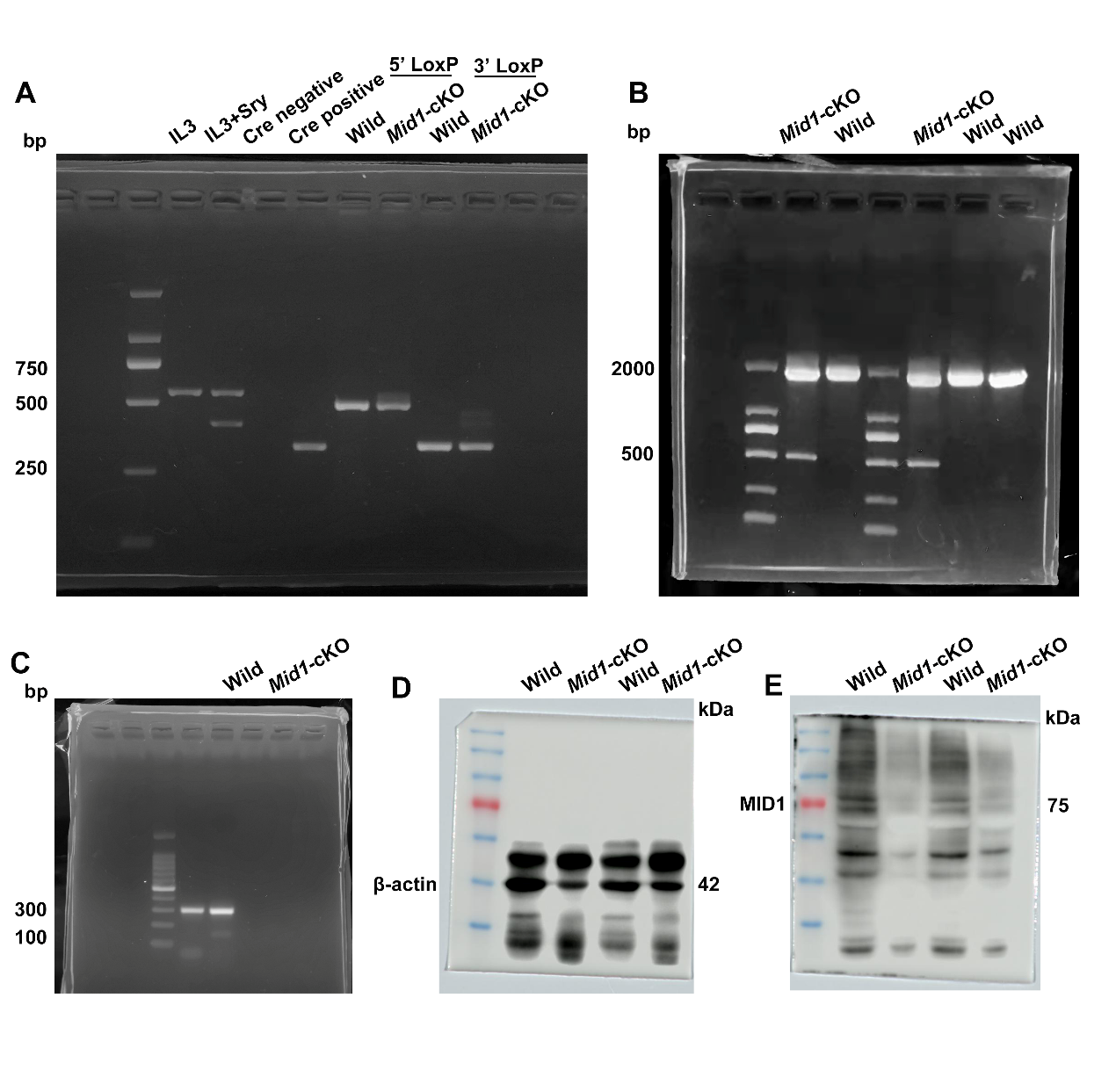


**Fig S4.** Original figures of gel and blot

A. PCR genotyping. Fragments of IL3 (544 bp) and Sry (402 bp, male-specific) were amplified for determination the gender of the mice. A 350 bp Cre replicon was amplified from the expression cassette of the Cre recombinase. Allele-specific PCR amplified 499 bp wild-type and 557 bp loxP fragments at the 5’ end loxP site to identify the F1 heterozygous. Allele-specific PCR amplified 344 bp wide-type and 466 bp loxP amplicons at the 3’ end loxP site to determine the heterozygosity at this locus.

B. PCR testing for identifying the deletion of *Mid1* exon 5. A 1721 bp wild-type and a 511 bp knockout fragments were amplified using genomic DNA of *Mid1*-cKO mice.

C. RT-PCR using the cDNA of a *Mid1*-cKO male mice, showing a 173 bp amplicon of the transcript from *Mid1* mutant allele in target tissue and a 310 bp amplicon of the transcript from intact *Mid1* allele.

D and E. Western blot detected a 75-kDa MID1 protein in wild-type male mice and a reduced expression in *Mid1*-cKO male mice. Protein levels were normalized with respect to the level of β-actin expression.
