# Supplementary material for: Morphometric analysis of the size-adjusted linear dimensions of the skull landmarks revealed craniofacial dysmorphology in Mid1-cKO mice
Source: BMC Genomics. 2023 Feb 9;24:68. doi: 10.1186/s12864-023-09162-2 (PMC9912615; doi:10.1186/s12864-023-09162-2)
Supplement: Supplementary file 10 — Additional file 10: Table S6. Breeding schemes. [file 12864_2023_9162_MOESM10_ESM.docx]

**Table S6. Breeding schemes.**

| Maternal  (*Mid1^flox/+^*) | Paternal (*Wnt1-Cre*) | | | |
| --- | --- | --- | --- | --- |
|  | A | B | C | D |
| F1-1 | *Mid1^flox/y^* (1)  *Mid1^flox/y^; Wnt1-Cre* (2)  *Mid1^+/+^; Wnt1-Cre* (2)  *Mid1^+/+^*(1)  *Mid1^flox/+^*(2) |  |  |  |
| F1-2 |  | *Mid1^flox/y^* (1)  *Mid1^flox/+^* (1)  *Mid1^+/+^; Wnt1-Cre* (2) |  |  |
| F1-3 |  | *Mid1^flox/y^* (2)  *Mid1^+/y^; Wnt1-Cre* (2)  *Mid1^+/+^*(2)  *Mid1^flox/+^; Wnt1-Cre* (1)  *Mid1^+/+^; Wnt1-Cre* (1)  *Mid1^flox/+^*(1) |  |  |
| F1-4 |  | *Mid1^flox/y^; Wnt1-Cre* (1)  *Mid1^flox/+^; Wnt1-Cre* (2)  *Mid1^flox/+^*(1)  *Mid1^+/+^*(1)  *Mid1^+/+^; Wnt1-Cre* (1) |  |  |
| F1-5 |  | *Mid1^flox/y^* (1)  *Mid1^+/+^*(1)  *Mid1^+/+^; Wnt1-Cre* (1)  *Mid1^flox/+^; Wnt1-Cre* (3) |  |  |
| F1-6 |  | *Mid1^flox/y^; Wnt1-Cre* (3)  *Mid1^flox/y^* (2)  *Mid1^flox/+^*(3)  *Mid1^flox/+^; Wnt1-Cre* (2) |  |  |
| F1-7 |  |  |  | *Mid1^+/y^; Wnt1-Cre* (1)  *Mid1^+/+^* (2)  *Mid1^flox/+^*(1) |
| F1-8 |  |  |  | *Mid1^+/y^; Wnt1-Cre* (1)  *Mid1^+/+^* (1)  *Mid1^+/+^; Wnt1-Cre* (1)  *Mid1^flox/+^; Wnt1-Cre* (1) |
| F1-9 |  |  | *Mid1^+/y^; Wnt1-Cre* (2)  *Mid1^+/y^* (1)  *Mid1^flox/y^* (1)  *Mid1^flox/+^; Wnt1-Cre* (1)  *Mid1^+/+^; Wnt1-Cre* (1) |  |
