# Supplementary material for: Morphometric analysis of the size-adjusted linear dimensions of the skull landmarks revealed craniofacial dysmorphology in Mid1-cKO mice
Source: BMC Genomics. 2023 Feb 9;24:68. doi: 10.1186/s12864-023-09162-2 (PMC9912615; doi:10.1186/s12864-023-09162-2)
Supplement: Supplementary file 11 — Additional file 11: Table S7. Skull landmarks used in morphometricanalysis. [file 12864_2023_9162_MOESM11_ESM.docx]

**Table S7 Skull landmarks used in morphometric analysis**

| Skull region | L/RMs No. | Abbreviation | Description |
| --- | --- | --- | --- |
| Facial skeleton | L/R1 | lnsla | Most antero-medial point of the nasal bone, left/right side |
|  | L/R2 | lnslp | Most postero-medial point of the nasal bone, left/right side |
|  | L/R3 | lflac | Intersection of frontal process of maxilla with frontal and lacrimal bones, left/right side. (Taken on the lump on the frontal process of the maxilla in P0) |
|  | L/R4 | lzya | Intersection of zygoma with zygomatic process of maxillar, taken on maxilla, left/right side |
|  | L/R5 | lzygo | Intersection of zygoma with zygomatic process of maxillar, taken on zygoma, left/right side |
|  | L/R6 | lzyt | Intersection of zygoma with zygomatic process of temporal, taken on zygoma, left/right side |
|  | L/R7 | lnasapl | Most supero-anterior point of the premaxilla accounting for the lateral part of the nasal aperture, left/right side |
|  | L/R8 | lnasapi | Most infero-medial point on the alveolar bone defining the lower part of the nasal aperture, left/right​ |
|  | L/R9 | lmaxi | The midline point on the premaxilla between the incisor and the nasal cavity just anterior of the incisive foramen, left/right side |
|  | L/R10 | laalf | Most anterior point of the anterior palatine foramen, left/right side |
|  | L/R11 | lpmx | Most infero-lateral point of the premaxillary-maxillary suture, taken on premaxilla, left/right side |
|  | L/R12 | lpalf | Most posterior point of the anterior palatine foramen, left/right side |
|  | L/R13 | lpppt | Most posterior projecting point on the turbinate, left/right side |
|  | L/R14 | lva | Most posterior point on the left/right ala of the vomer |
|  | L/R15 | lfppm | Most supero-posterior point of the premaxilla accounting for the lateral part of the nasal aperture, left/right side of the lateral part of the nasal aperture |
|  | L/R16 | lnsll | Most postero-lateral point of the nasal bone, left/right side |
|  | L/R17 | liohs | Most superior point of the infraorbital hiatus, left/right side |
|  | L/R18 | liohi | Most inferior point of the infraorbital hiatus, left/right side |
|  | L/R19 | liohd | Most distal point of the infraorbital hiatus, left/right side |
|  | L/R20 | lmma | Posterior lateral point on the maxillary portion of the medial alveolus, left/right side |
|  | L/R21 | lpmp | Most posteromedial point on the posterior palatine plate, left/right side |
|  | L/R22 | lalp | Most anterolateral point on the palatine plate, left/right side |
|  | L/R23 | lamp | Most anteromedial point on the posterior palatine plate, left/right side |
|  | L/R24 | lplpp | Most posterolateral point on the palatine plate, left/right side |
|  | L/R25 | lpns | Most antero-lateral indentation at the posterior edge of the palatine plate, left/right side |
|  | L/R26 | lsqu | Most superior point on the squamous temporal, intersection of the coronal suture, left/right side |
|  | L/R27 | lzptb | Basis of the zygomatic process of the temporal, left/right side |
|  | L/R28 | lpsq | Most posterior point on the posterior extension of the forming squamosal, left/right side |
|  | L/R29 | lipmax | Most inferior point on the margin of the max-premax suture, taken on premaxilla, left/right side |
|  | M1 | ethma | Anterior most point on the body of the vomer, taken on the ventral surface |
|  | M2 | ethmp | Posterior most point on the body of the vomer, taken on the ventral surface between the alae |
| Cranial vault | L/R30 | lalf | Most anteromedial point on the frontal bone, left/right side |
|  | L/R31 | loptcan | Most supero-posterior point of the optic canal, left/right side​ |
|  | L/R32 | laif | Most anteroinferior point on the frontal bone, left/right side |
|  | L/R33 | lfpl | Most lateral intersection of the frontal and parietal bones, taken on the frontal, left/right side |
|  | L/R34 | lpfl | Most lateral intersection of the frontal and parietal bones, taken on the parietal, left/right side |
|  | L/R35 | lfpi | Most medial intersection of the frontal and parietal bones, taken on the frontal, left/right side |
|  | L/R36 | lpfm | Most medial intersection of the frontal and parietal bones, taken on the parietal, left/right side |
|  | L/R37 | lpto | Most postero-medial point on the parietal, left/right side |
|  | L/R38 | lpip | Most postero-inferior point on the parietal, left/right side |
|  | L/R39 | locc | Most infero-lateral point on the squamous occipital, left/right side |
|  | L/R40 | loci | The superior posterior point on the ectocranial surface of occipital lateralis on the foramen magnum, left/right side |
|  | L/R41 | loccond | Antero inferior elevation of the lateral inferior process of the occipital condyle, left/right side |
|  | M3 | intpar | Most anterior point on the ectocranial surface of the interparietal on the midsagittal plane |
|  | M4 | opi | Mid-point on the posterior margin of the foramen magnum, taken on squamous occipital |
| Cranial base | L/R42 | lpsh | Most anterior point on the anterior projection on the presphenoid, left/right side |
|  | L/R43 | lsyn | Most antero-lateral point on corner of the basioccipital, left/right side |
|  | L/R44 | lptyp | Most posterior tip of the medial pterygoid process, left/right side |
|  | L/R45 | latyp | Most anterior tip of the pterygoid process, left/right side |
|  | L/R46 | lasph | Postero-medial point of the inferior portion of the left alisphenoid, left side/right alisphenoid, right side |
|  | L/R47 | lcarcan | Most medial point of the carotid canal, left/right side |
|  | L/R48 | later | Most anterior tip of the ectotympanic ring, left/right side |
|  | L/R49 | lpter | Most posterior tip of the ectotympanic ring, left/right side |
|  | M5 | cpsh | Most anterior point of the indentation in the center of the presphenoid |
|  | M6 | bas | Mid-point on the anterior margin of the foramen magnum, taken on basioccipital |
|  | M7 | amsph | Most antero-medial point on the body of the sphenoid |
